# The mining and evolutionary investigation of *AP2/ERF* genes in pear (*Pyrus*)

**DOI:** 10.1186/s12870-018-1265-x

**Published:** 2018-03-20

**Authors:** Xiaolong Li, Shutian Tao, Shuwei Wei, Meiling Ming, Xiaosan Huang, Shaoling Zhang, Jun Wu

**Affiliations:** Centre of Pear Engineering Technology Research, State Key Laboratory of Crop Genetics and Germplasm Enhancement, Nanjing Agricultural University, Nanjing, 210095 China

**Keywords:** ERF family, Evolution, Expression pattern, Abiotic stress

## Abstract

**Background:**

In plants, *ERF* genes participate in a variety of regulatory pathways, such as plant growth and biotic and/or abiotic stress responses. Although the genome of Chinese white pear (‘Dangshansuli’) has been released, knowledge regarding the ERF family in pear, such as gene functions, evolutionary history and expression patterns, remains limited.

**Results:**

In our study, a total of 155 members of ERF families were identified in pear (*Pyrus bretschneideri*). The Ka and Ks values suggested that whole-genome duplication (WGD) and dispersed duplication have effectively contributed to the expansion of the pear ERF family. Gene structure and phylogeny analysis divided the *PbrERF* family into 12 groups, and their gene functions were predicted by comparative analysis. qRT-PCR was carried out to verify the relative expression levels of 7 genes in group III using wild and cultivated pear fruits at three key developmental stages. Wild samples had higher expression of these genes than cultivated samples, especially at the enlarged fruit stage. The transcriptome data of pear seedlings subjected to dehydration treatment further revealed that 4 of the 7 genes responded to drought conditions.

**Conclusion:**

The *AP2/ERF* gene family is greatly expanded in pear. Comparative analysis revealed the probability of *ERF* genes performing functional roles in multiple pathways. Expression analysis at different stages of pear fruit development in wild and cultivated samples indicated that genes in group III might be involved in abiotic and/or biotic stresses. Further transcriptome data on seedlings subjected to drought treatment verified the potential role of *ERF* genes in stress response. These results will provide a valuable reference for understanding the function and evolution of the ERF family in higher plants.

**Electronic supplementary material:**

The online version of this article (10.1186/s12870-018-1265-x) contains supplementary material, which is available to authorized users.

## Background

The *AP2/ERF* superfamily, plant-specific transcription factors, is characterized by a large number of members with great functional divergence. Members contain one or several conserved *AP2/ERF* domains consisting of approximately 60 to 70 amino acids in the DNA-binding region. Based on the number of conserved domains, the superfamily can be classified into three types, the *AP2, ERF,* and *RAV* gene families [1]. In the *AP2* family, each protein sequence contains two *AP2/ERF* domains. In the *ERF* family, only a single *AP2/ERF* domain occurs in each protein sequence, while members of the *RAV* family contain not only an *AP2/ERF* domain but also a B3 domain—a conserved DNA-binding domain present in other plant-specific transcription factors. Furthermore, the *ERF* family can be divided into two major subfamilies, *CBF/DREB* and *ERF* [2]. In the *ERF* subfamily, the conserved nucleotide sequence AGCCGCC of the GCC-box [3] is found in the promoter regions of pathogenesis-related (PR) genes, which regulate gene expression in disease resistance response pathways [4]. In the *DREB* family, the conserved binding sequence CCGAC typically binds to cis-acting elements and participates in responses to abiotic stress (cold and drought) and in the regulation of plant hormones such as ABA and ethylene [5, 6] via the regulation of gene expression. Previous studies have shown that *AP2* genes might regulate plant organ growth and development pathways, such as flower development and the determination of seed size [7–9]. Genes in the *RAV* family were predicted to participate in the response to ethylene [10] and to biotic and/or abiotic stresses [11, 12].

The release of high-throughout sequencing data has enabled identification and analysis of gene families at the genome-wide level. To date, whole-genome identification and analysis of the *ERF* gene family have been performed in many species, such as castor bean [13], *Arabidopsis* [14], poplar [15], grape [16], rice [17], wheat [18], cucumber [19], and soybean [20]. Pear, one of the most important fruits, in the Rosaceae family, is widely distributed worldwide. However, knowledge of the *ERF* family in pear remains severely limited. The recent completion of pear genome sequencing and assembly [21] provides us an opportunity to identify and dissect the *AP2/ERF* family. This investigation will provide insights into the function and evolution of the *AP2/ERF* family in pear.

In our study, we used the genome sequence data of pear to explore the *AP2/ERF* family on the whole-genome level. A total of 191 unigenes were identified as candidate members of the *AP2/ERF* family, including 155 *ERF* unigenes, 26 *AP2* unigenes, and nine *RAV* unigenes. Phylogenetics, gene structure, and predicted function were characterized for the *AP2/ERF* family, as well as gene expression patterns. These results build a solid foundation for the future exploration of *ERF* gene functions in pear.

## Methods

### Identification of AP2/ERF gene family

Based on the pear genome project (http://peargenome.njau.edu.cn/) [21], we downloaded all protein sequences to identify members of the *AP2/ERF* gene family. The *Arabidopsis AP2/ERF* genes were identified in a previous study [22], and their amino acid sequences were downloaded from the Plant Transcription Factor Database (PlantTFDB) (http://planttfdb.cbi.pku.edu.cn). We used two approaches to obtain members of the *ERF* family in *Pyrus bretschneideri*. First, a Hidden Markov Model search (HMMsearch) was performed using the HMM profile with the AP2 domain (PF00847). Second, a BLASTP alignment against all pear protein sequences was used to perform an extensive search for candidate *AP2/ERF* genes using *ERF* protein sequences from tobacco and *Arabidopsis thaliana* as queries. For all sequences searched using these two methods, we first removed the redundant sequences and incomplete sequences. Then, we used the SMART tool (http://smart.embl-heidelberg.de/) and the InterProScan tool (http://www.ebi.ac.uk/Tools/pfa/iprscan/) to detect the presence or absence of the AP2/ERF domain in the candidate protein sequences. Protein sequences with one or more AP2/ERF domains were identified and retained as putative members of the *AP2/ERF* family for subsequent analyses. The location information on each pear *AP2/ERF* gene was obtained from the pear genome database. Then, the data were displayed by plotting a graph using Circos software [23].

### Phylogenetics, gene structure and motif analyses

An un-rooted phylogenetic tree was constructed using MEGA6.0 [24] with neighbor-joining (NJ) criteria and verified using the maximum likelihood (ML) method, and 1000 bootstrap replicates were performed based on multiple alignments of the full-length amino acid (AA) sequences of all AP2/ERF genes in pear and *Arabidopsis* using ClustalW [25]. Based on the alignments of CDS sequences with the corresponding full-length genomic sequences, the gene structures of the *AP2/ERF* family were displayed using an online website: Gene Structure Display Server (GSDS) (http://gsds.cbi.pku.edu.cn/). Moreover, conserved motifs were detected in pear *AP2/ERF* family members using the motif analysis tool MEME (http://meme-suite.org/tools/meme) with the default parameters except for two: motif site distribution, any number of repetitions; maximum number of motifs, 30.

### Synteny analysis and calculation of Ka and Ks values

We used a method distinct from that used in the Genome Duplication Database (PGDD) (http://chibba.agtec.uga.edu/duplication/) [26] to perform the synteny analysis. First, to identify the candidate homologous gene pairs (E < 1e-5, top 5 matches), a BLASTP alignment was carried out across the whole genome. The potential homologous gene pairs identified were then loaded into the software MCScanX with the default parameters [27, 28] to identify syntenic chains. We also used MCScanX to further distinguish the WGD/segmental, dispersed proximal, and tandem duplication event types in the *ERF* gene family.

Furthermore, candidate homologous gene pairs identified from the same synteny block were used as the input for the software KaKs_Calculator 2.0 [29] to calculate the Ka and Ks values. The software parameters were set as follows: YN as the Method (−m) and Standard Code as the Genetic code table (−c). Then, we used a python script written in-house to obtain the Ka and Ks values of the identified syntenic genes.

### Gene expression analyses

The RNA-Seq data on ‘Dangshansuli’ obtained from our previous study [21] and downloaded from the pear genome website (http://peargenome.njau.edu.cn) were used to analyze the expression of *PbrAP2/ERF* genes at six different developmental stages of pear fruit: S1 (15 DAF), S2 (36 DAF), S3 (80 DAF), S4 (110 DAF), S5 (145 DAF), and S6 (167 DAF). The RNA-Seq data from five different dehydration treatments of pear seedlings obtained in the previous study were also downloaded [30]. The heatmaps were plotted in R using the heatmap.2 function based on the logarithmically (log2) transformed reads per kilobase per million (RPKM) values of each *AP2/ERF* gene.

### RNA extraction and cDNA synthesis

We collected six pear accessions including three wild accessions of *P. pyrifolia*, ‘Matanggengzi’ (‘MTGZ’), ‘Baitanggengzi’ (‘BTGZ’), and ‘Tiantanggengzi’ (‘TTGZ’), and three cultivated accessions, ‘Huanghuali’ (‘HH’), ‘Lipuxueli’ (‘LPXL’), and ‘Liuchengfengshan’ (‘LCFS’)*,* at three fruit developmental stages (small fruit stage, 52 DAF; enlarged fruit stage, 94 DAF; mature fruit stage, 128 DAF) for qRT-PCR analysis. First, we mixed the pear fruit samples of the same developmental stages from wild or cultivated genotypes. Then, the Plant Total RNA Isolation Kit Plus (FOREGENE Co. Ltd.) was used to extract the total RNA from the mixed samples of pear fruit. In the process, we carried out an improved step that was proposed in our previous study [31]. To obtain higher-quality RNA from pear fruit at the late stage of development, with high water content, less water (40 μl) was used to elute the RNA from the filtration column. Then, the total RNA was adjusted to the same concentration, and based on the adjusted RNA, first-strand cDNA was synthesized using TransScript One-Step gDNA Removal and cDNA Synthesis SuperMix (TransGen Biotech Co. Ltd.).

### Quantitative real-time PCR analysis (qRT-PCR)

Seven pairs of the most reliable primers (Additional file 1) were designed to amplify the seven candidate gene sequences using online software from NCBI (National Center for Biotechnology Information) (https://www.ncbi.nlm.nih.gov/tools/primer-blast/). According to the method described in a previous study [31], the LightCycler 480 SYBR GREEN I Master (Roche) was used to perform the qRT-PCR analysis. A 20 μl mixed reaction system was constructed, each containing 100 ng of template cDNA, 0.5 μM of each primer and 10 μl of LightCycler 480 SYBR GREEN I Master. All reactions were carried out in 96-well plates with four replicates for each cDNA sample. We set the qRT-PCR conditions as follows: first 5 min at 95 °C for pre-incubation, 55 cycles at 95 °C for 3 s, 60 °C for 10 s, and 72 °C for 30 s, and then 3 min at 72 °C for extension. Finally, the step of fluorescence signal data collection was carried out at 60 °C. *Pyrus Actin* (accession No. AF386514) and *Pyrus GAPDH* were used as the internal control genes. The average threshold cycle (Ct) of each cDNA sample was calculated using the running results displayed on the computer. Meanwhile, the relative expression levels of seven genes were calculated using the 2^-∆∆Ct^ method described in a previous study [32].

## Results

### Identification of AP2/ERF genes in pear genome

**Fig. 1:**
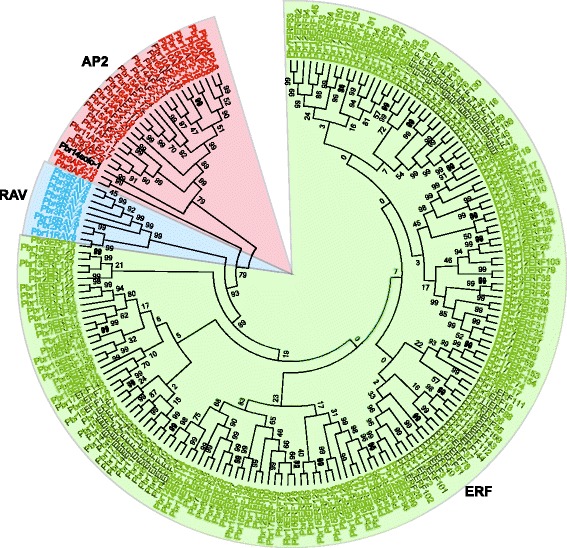
Phylogenetic tree of 191 *AP2/ERF* family proteins in pear**.** Red font represents the *AP2* group; blue represents the *RAV* group; green represents the *ERF* group; black represents Soloist

**Fig. 2:**
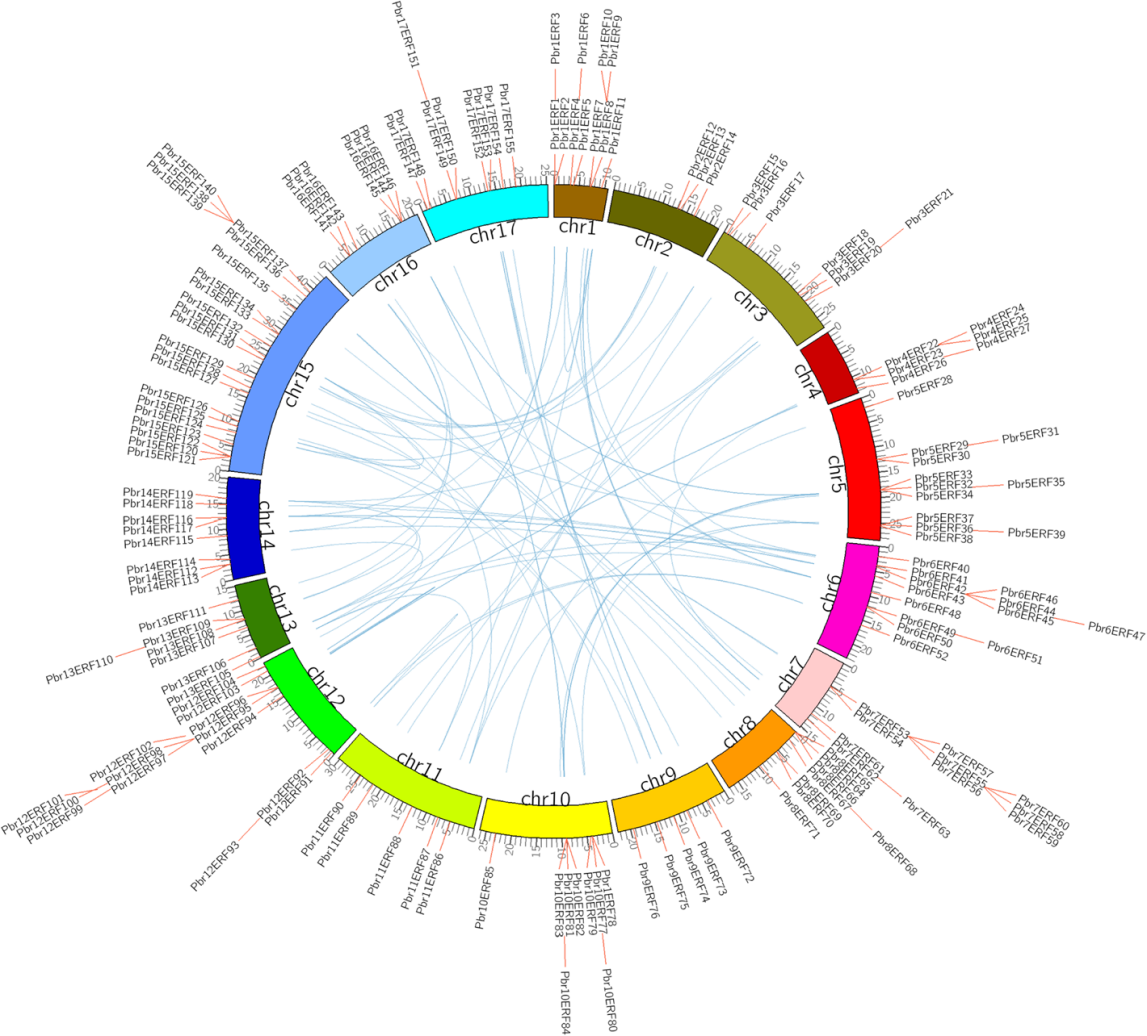
Distribution and collinearity of the AP2/ERF members in the pear genome. Red lines mark the positions of genes on chromosomes. Blue lines indicate collinearity relationships among genes

Members of the *AP2/ERF* family were searched for in the Chinese white pear (*P. bretschneideri*) genome using two strategies: Hidden Markov Model search (HMMsearch) using the HMM profile (PF00847) of the AP2 domain and BLASTP search using *ERF* proteins from tobacco and *Arabidopsis thaliana* as queries. A total of 240 sequences were matched as candidate *AP2/ERF* genes across the whole genome. Among these candidates, we removed 41 *AP2/ERF* genes located in unanchored scaffolds, as well as two (*Pbr039133.1* and *Pbr002083.1*) containing one Amb_all domain and one X8 domain, respectively. Meanwhile, six genes were removed because of incomplete domains. Consequently, 191 non-redundant and complete *AP2/ERF* genes were surveyed in our study. Based on their domain structures, the *AP2/ERF* superfamily genes were classified into three families: *ERF* (one AP2 domain), *AP2* (two AP2 domains), and *RAV* (one AP2 and one B3 domain). Of these 191 genes, 22 genes were assigned to the *AP2* gene family and encode proteins containing two repeated AP2/ERF domains. Interestingly, although the gene *Pbr038562.1* contains two AP2/ERF domains, it is more closely related to the ERF type (Fig. 1). Eight genes were assigned to the *RAV* gene family and were predicted to encode one AP2/ERF domain and one B3 domain. Meanwhile, 161 genes encode proteins containing only a single AP2/ERF domain. Of these 161 genes, 155 were assigned to the *ERF* family. Of the remaining six genes, *Pbr033071.1* (*Pbr2AP2–3*), *Pbr025458.1* (*Pbr10AP2–14*), *Pbr023949.1* (*Pbr1AP2–1*), and *Pbr022083.1* (*Pbr1AP2–2*) also encode a single AP2/ERF domain, but are distinct from the ERF family and instead clustered into the AP2 family. Therefore, these six genes were further analyzed as *AP2* genes (Additional file 2). Similarly, the gene *Pbr030666.1* (*Pbr9RAV7*) encodes an AP2/ERF domain but is more similar to the RAV family. Finally, the gene *Pbr002042.1* (*Pbr14solo-1*) was assigned as a soloist, with low homology to other *AP2/ERF* genes, although it includes an AP2/ERF-like domain sequence. A previous study showed that more *AP2/ERF* genes were identified in apple, including 51 *AP2* genes, six *RAV* genes, 195 *ERF* genes, and seven soloists [33], supporting great expansion of the *AP2/ERF* family in both pear and apple. To distinguish each family member, we named these genes according to classification of the family and the order of the chromosome locations (Additional file 2). For example, genes in the *ERF* family were named from *Pbr1ERF1* to *Pbr17ERF155*. Furthermore, the location information of each gene was used to display the distribution of the *PbrERF* genes in the pear genome. The results showed that all 155 *PbrERF* genes were distributed on chromosomes 1 through 17 as shown in Fig. 2.

### Phylogenetics, gene structure, and conserved motif analyses

**Fig. 3:**
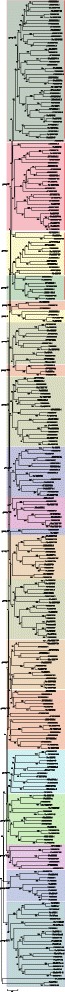
Phylogenetic trees of pear and *Arabidopsis AP2/ERF* proteins. The full-length sequences of all *AP2/ERF* proteins were aligned using ClustalX, and the phylogenetic tree was generated using MEGA 6.0 with the NJ method

**Fig. 4:**
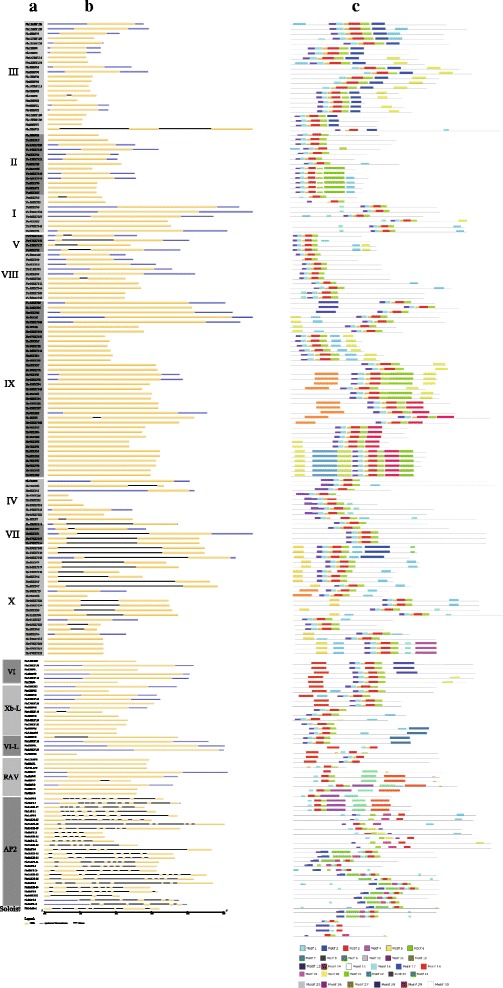
Analyses of gene structure and conserved motifs within each *AP2/ERF* clade in pear. **a** Clades identified in *AP2/ERF* family. **b** Exon/intron structures of pear *AP2/ERF* genes. Yellow boxes represent the exons. Blue boxes represent UTRs. Black lines connecting two exons indicate introns. **c** The distribution of conserved motifs within each *AP2/ERF* gene. Boxes in different colors represent different conserved motifs, and their relative positions are displayed

To explore the evolutionary relationships among the 191 *AP2/ERF* genes identified in pear, we constructed a phylogenetic tree using MEGA 6.0 based on multiple sequence alignments of 122 *Arabidopsis ERF* genes obtained from a previous study [7] and 191 pear *AP2/ERF* genes. The resulting phylogenetic tree allowed us to split the *AP2/ERF* family into 12 distinct clades from I to Xb-L, which were strongly supported by bootstrap values as shown in Fig. 3. Based on the classification of the *AP2/ERF* family in *Arabidopsis thaliana* [7], the *DREB* subfamily and the *ERF* subfamily were clearly separated, consisting of clades I-IV and clades V-Xb-L, respectively. In addition, the *AP2* subfamily in pear contained 26 members; the clade *RAV* subfamily included nine members; and Soloist was separated (Table 1; Fig. 4a). Further, we found that most of the clades and subclades consisted of genes from both the pear and *Arabidopsis AP2/ERF* families, which indicated that the *AP2/ERF* genes are homologous and evolved from a common ancestor between pear and *Arabidopsis*.

**Table 1:** Summary of the structure of the *AP2/ERF* superfamily in pear, Chinese plum, *Arabidopsis*, grape, rice, cucumber, poplar, and soybean

Family	Subfamily	Group	Pear	Arabidopsis	*Vitis vinifera*	Poplar	Soybean	Chinese plum	Rice	Cucumber
AP2			26	18	20	26	26	20	29	20
ERF		Total	155	122	122	169	120	90	145	103
	DREB	Total	53	57	40	66	43	35	58	42
		I	8	10	5	5	9	5	9	5
		II	13	15	8	20	7	4	16	10
		III	23	23	22	35	21	20	27	20
		IV	9	9	5	6	6	6	6	7
	ERF	Total	101	65	82	103	77	55	87	61
		V	4	5	11	10	7	10	8	15
		VI	6	8	5	11	5	5	6	8
		VII	5	5	3	6	10	3	15	3
		VIII	10	15	11	17	12	10	15	11
		IX	37	17	40	42	21	18	18	16
		X	23	8	10	9	6	7	12	8
		VI-L	4	4	2	4	7	2	3	
		Xb-L	12	3	0	4	7	0	10	
RAV			9	6	6	6	2	5	5	4
Soloist			1	1	1	1		1	1	4
Total			191	147	149	202	148	116	180	131

Gene structure analyses provided further evidence to support the phylogenetic topology groupings of gene families and showed that members of the *AP2* family had intron numbers from six to ten, eight members in the *RAV* family had lost all introns, and *Pbr9RAV7* had one. Most members of the *ERF* subfamily had only one exon and two UTR regions, but most members of groups V, VII, and X had two exons and one intron. Meanwhile, the *Pbr14solo-1* gene contained seven introns and eight exons. Further, intron positions were found to be conserved in the *AP2* family, although the number of introns varied. Likewise, most of the *ERF* family members shared the same or similar intron patterns, with most introns located in the AP2/ERF domain regions, as shown in Fig. 4b. In general, many conserved motifs could be detected in the protein sequences of transcription factors and may participate in activating gene expression as potential DNA-binding sites. In previous studies, diverse conserved motifs have been identified in rice and *Arabidopsis,* and their functions have been investigated [15, 22]. To characterize potential conserved motifs from the amino acid sequences of the *AP2/ERF* members, we used the Multiple Em for Motif Elicitation (MEME) tool [34] to analyze the 191 *AP2/ERF* amino acid sequences in pear. A total of 30 conserved motifs were detected and named 1–30 (see Fig. 4c). The results showed that most members of each group contained the same motifs. The gene structures provided reliable evidence to support and validate previous phylogenetic groupings.

### Function prediction of each group in the PbrERF gene family

A previous study predicted the function of each group of the *ERF* family in *Arabidopsis* [22]. In our study, phylogenetic analysis allowed us to identify putative orthologous and paralogous *ERF* genes in pear and *Arabidopsis*. In general, homologous genes share similar gene structures and are clustered in the same clades, in which the genes possess similar functions. To predict the gene functions of each group in the pear *ERF* family, we constructed a phylogenetic tree via comparative genomics to define the ERF groups of orthologs from pear and *Arabidopsis* (Fig. 3). In group I, although the functions of all *Arabidopsis ERF* genes are unknown, the *At1g78080* protein and the *WXP1* protein share conserved motifs, and the overexpression of gene *WXP1* has been reported as an activator in the regulation of wax synthesis in *Medicago truncatula* [35]. Therefore, we predicted that *ERF* genes of group I might play an important role in wax accumulation in pear as well. The functions of *Arabidopsis* group III *ERF* genes have been extensively studied and include potentially crucial functional roles in the response to cold, drought, and salt stresses [36–38]. Although the functions of group III protein in pear are unknown, these proteins may also participate in abiotic stress responses as transcriptional activators, based on the similar and conserved motif regions. In group IV, *DREB2A* (*At5g05410*) and *DREB2B* (*At3g11020*) have been reported to regulate DRE-mediated transcription as transcription factors [37], and the gene *AtERF#052* (*ABI4*) participated in ABA signaling [39] and sugar response pathways [40, 41]. In group V, overexpression of the gene *WIN1/SHN1* (*At1g15360*) contributed to the accumulation of leaf epidermal wax [42, 43], and genes *SHN2* (*At5g11190*) and *SHN3* (*At5g25390*) had similar functions. All genes of group V shared a conserved motif structure. Therefore, other pear *ERF* genes of group V may reasonably be hypothesized to play important roles in the pathway of wax accumulation as well. In addition, the fewer *PbrERF* genes in group V in pear than apple [33] (4 genes vs. 19 genes) might be due to its functional redundancy in controlling wax accumulation. The proteins in group VI share conserved motifs in the N-terminal region. In previous studies, the tobacco *Tsi1* protein [44] and tomato *Pti6* proteins [45] exhibited the similar characteristic gene structure features to members of group VI, and the genes *Tsi1* and *Pti6* have been reported to respond to abiotic and/or biotic stresses by regulating the expression of key genes in the pathway. In the group VI-L, all proteins also have the two conserved motifs that characterize group VI. In group VII, the gene *AtEBP* (*At3g16770*) was identified as a key gene interacting with the *bZIP* transcription factor *OBF4* in vitro, although the function of this interaction remained unknown [46]. In addition, gene structure analyses showed that all genes in group VII have only a single intron and share completely consistent motifs in the 5′-flanking region of the AP2/ERF domain (Fig. 4). In group VIII, *AtERF4* (*At3g15210*) and *AtERF7* (*At3g20310*) were shown to negatively regulate gene expression in response to ABA, jasmonic acid, and ethylene [47–49]. Moreover, the genes *LEP* (*At5g13910*) [50] and *ESR1/DRN* (*At1g12980*) [51, 52] participate in the regulation of organ differentiation and plant development. Thus, we hypothesized that other pear genes in group VIII might have similar functions. In group X, *Arabidopsis ABR1* (*At5g64750*) was reported in a previous study to participate in ABA response as a repressor, and knockout of the gene *ABR1* resulted in an excitatory response to ABA in root growth and seed germination processes [53].

### Gene duplication and synteny analyses in the PbrERF gene family

Five gene duplication types can be detected in a genome, including whole-genome duplication (WGD), singleton duplication, tandem duplication, proximal duplication, and dispersed duplication. These duplication events are the major driving force in the expansion of gene families [54]. Therefore, to explore the origins of duplicate genes in the *PbrERF* gene family, we dissected the duplication type of each member of the *PbrERF* family by using the software package MCScanX. Each gene in the *PbrERF* family was assigned to one of the five gene duplication types. Among them, 66.45% (103) of the pear *PbrERF* genes were retained from WGD or segmental duplication events, compared to only 5.16% (8) from tandem, 19.35% (30) from dispersed, and 9.03% (14) from proximal duplication events (Additional file 3).

To further provide additional evidence for a WGD event as the major force contributing to the expansion of the *PbrERF* gene family, a method developed from the one used in the Plant Genome Duplication Database (PGDD) was used to identify synteny blocks across the whole pear genome. In our analysis, a total of 75 duplicated gene pairs (Additional file 4) were found in the *PbrERF* gene family. The similarity for each pair ranged from 30.22%~ 100.00%. Meanwhile, considering that orthologs often retain equivalent functions over the course of evolution, we examined the orthologous relationships of *ERF* genes between pear and *Arabidopsis* using the same method. A total of 84 genes from pear, with the exception of Soloist, have one or several putative orthologs in *Arabidopsis* (Additional file 5). Meanwhile, all of them were subdivided into the same group as their orthologs in *Arabidopsis*, which further supported the results of the phylogenetic analysis. Among these 84 genes, eight belong to the *AP2* family, 75 to the *ERF* family and only one to the *RAV* family. A previous study reported no *Arabidopsis* orthologs for the *RAV* family genes in Chinese plum [55]. Synteny analysis can be effectively used to provide strong support for putative paralogous or orthologous genes found through phylogenetic analyses.

### Estimation of dates and driving forces of evolution

**Fig. 5:**
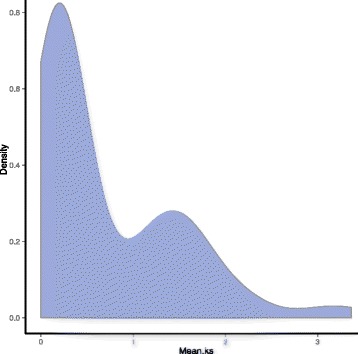
Distribution of mean Ks values of 75 pairs of *PbrMYB* genes. The x-axis represents the mean Ks value; y-axis represents the density of the distribution. The data were plotted using R

The synonymous substitution rate per site, that is, the Ks value, is usually used to estimate the evolutionary dates of WGD events. Previous studies have reported that the pear genome has undergone two WGD events: an ancient WGD (Ks ~ 1.5–1.8), which was inferred to have occurred ~ 140 MYA [56], and a recent WGD (Ks ~ 0.15–0.3), which was inferred to have occurred 30–45 MYA [21]. Therefore, we estimated the dates of the expansion of the *PbrERF* family by calculating the Ks value in our study. The mean Ks values of the *PbrERF* gene pairs duplicated in the WGD event in the syntenic region, ranging from 0.01 to 3.37, are shown in Additional file 4. Furthermore, 65 (86.67%) pairs of duplicated genes were distributed at the two Ks value peaks (Fig. 5). Thus, these duplicated gene pairs may have arisen from the same recent (30–45 MYA) and ancient (~ 140 MYA) WGDs, which led to the expansion of the *PbrERF* gene family.

Positive selection (Darwinian selection) drives the evolution of animals and plants via accumulation of new advantageous mutations, which are then spread throughout the population. In contrast, negative selection, that is, purifying selection, is an evolutionary process to remove deleterious mutations [57]. To further determine whether one or both selection processes have driven the evolution of the *PbrERF* family in pear, the Ka values (nonsynonymous substitutions per site) and Ka/Ks ratio of homologs were also calculated using coding sequences (CDS) of genes in the *PbrERF* family. The direction and magnitude of selection could be measured using the Ka/Ks ratio: values less than one indicate negative selection, equal to one indicates neutral selection, and greater than one indicates positive selection [58]. In these analyses, all 75 gene pairs possessed Ka/Ks ratios less than one except for the gene pair *Pbr017391.1-Pbr030208.1*, whose Ka/Ks ratio was greater than one, implying that purifying selection has driven *PbrERF* family evolution as the primary force. Meanwhile, we also proposed that the genes *Pbr017391.1* and *Pbr030208.1* might play important roles related to the evolution of plant phenotypic traits such as fruit size and sugar or acid content.

### Expression analyses of AP2/ERF genes in pear

**Fig. 6:**
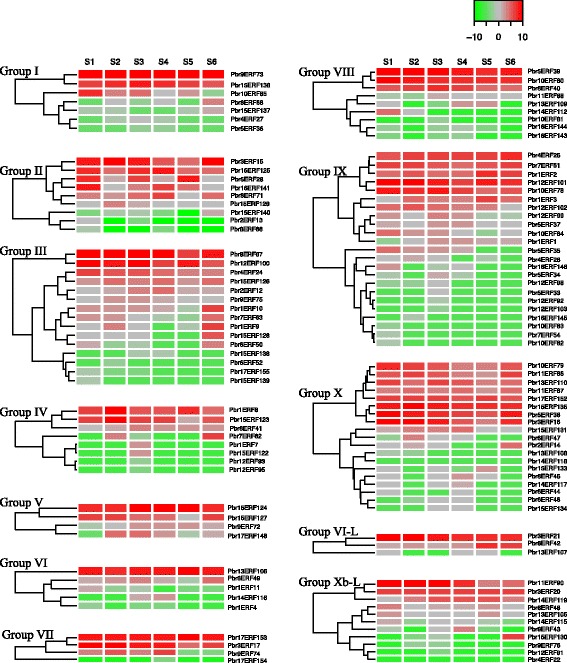
Expression heatmap of pear *ERF* family genes at six developmental stages of fruit. The expression levels of *AP2/ERF* genes were measured by RNA-Seq analysis at six different stages: 15 DAF (S1), 36 DAF (S2), 80 DAF (S3), 110 DAF (S4), 145 DAF (S5), and 167 DAF (S6). The *AP2/ERF* gene family was subdivided into 12 groups: I, II, III, IV, V, VI, VI-L, VII, VIII, IX, X, and Xb-L. The color scale at the top right represents RPKM normalized by log2. Light green indicates a low expression level, gray indicates a medium level, and red indicates a high level. The graph was plotted in R using the heatmap.2 function

**Fig. 7:**
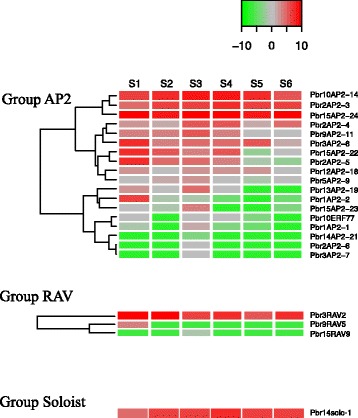
Expression heatmap of level of pear *AP2*, *RAV*, and *Soloist* genes. The expression levels of the *AP2*, *RAV*, and *Soloist* genes were measured by RNA-Seq analysis at six different stages: 15 DAF (S1), 36 DAF (S2), 80 DAF (S3), 110 DAF (S4), 145 DAF (S5), and 167 DAF (S6). The color scale at the top right represents RPKM normalized by log2. Light green indicates a low expression level, gray indicates a medium level, and red indicates a high level. The graph was plotted in R using the heatmap.2 function

Transcriptome sequencing (RNA-Seq) data from six different developmental stages of ‘Dangshansuli’ pear fruit were downloaded from our pear genome database (http://peargenome.njau.edu.cn) [21]. The expression patterns of 191 pear *AP2/ERF* genes are shown in Fig. 6 (*ERF* family) and Fig. 7 (*AP2* and *RAV* families, Soloist). Among these 191 genes, expression was not detected at any stage for 51 genes, and 140 genes were expressed in at least one pear fruit stage. Seventy-eight genes were detected at all six stages, although not all expression levels were high (Additional file 6).

The expression pattern of *PbrAP2/ERF* genes is varied, even within a single group. For example, group IX can be divided into three subgroups based on expression patterns. Five genes (*Pbr4ERF25*, *Pbr7ERF61*, *Pbr1ERF2*, *Pbr12ERF101*, and *Pbr10ERF78*) constitute a subgroup in which all genes were highly expressed in all six stages and are clustered together. Six genes (*Pbr1ERF3*, *Pbr12ERF102*, *Pbr12ERF99*, *Pbr5ERF37*, *Pbr10ERF84*, and *Pbr1ERF1*) constitute a second subgroup, in which all genes were expressed at low levels in each stage. However, the remaining genes can be clustered together and were expressed at low levels in one to four stages. Interestingly, in all groups, we could always detect one to six highly expressed genes at all fruit developmental stages.

**Fig. 8:**
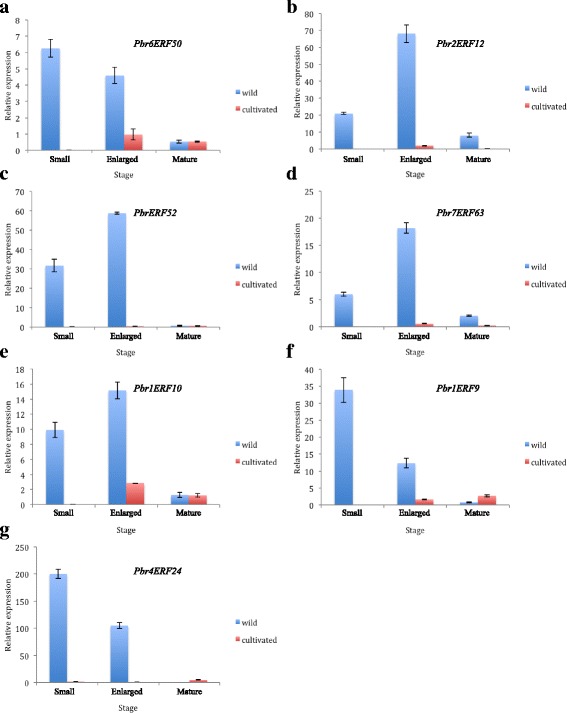
Relative expression levels of seven genes involved in the regulation of biotic and abiotic stresses. qRT-PCR analysis was performed to measure the expression levels of seven genes in subgroup III. Blue bars represent wild pear, and red bars represent cultivated pear. The x-axis represents the three stages, and the y-axis represent the relative expression levels of the genes, shown as the mean ± SD from three replications

Generally, wild plants have higher resistance than cultivated plants. To verify whether our functional cluster analysis was reliable, we selected seven genes of group III, predicted to be involved in abiotic and/or biotic stress response, for qRT-PCR analysis in wild and cultivated pears at three different developmental stages (small fruit stage, 52 Days After Flowering (DAF); enlarged fruit stage, 94 DAF; mature fruit stage, 128 DAF). The results indicated that these seven genes are significantly differently expressed in wild and cultivated pears, especially during the small and enlarged fruit stages (Fig. 8), and that the expression levels in wild pears are far higher than in cultivated pears. Accordingly, we could conclude that genes in group III regulated the abiotic stress response pathway via positive regulation and that resistance-related genes gradually lose their function during fruit ripening.

**Fig. 9:**
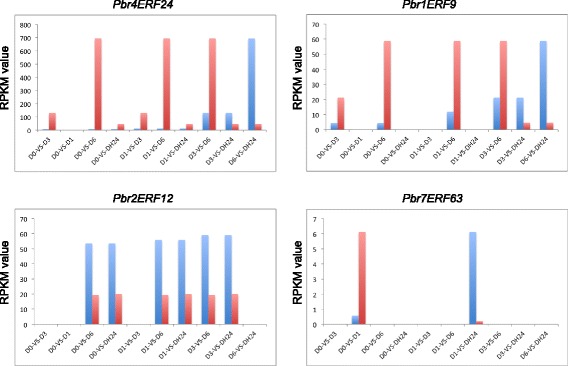
The RPKM values of stress-related *ERF* genes in subgroup III. Transcriptome data from dehydration treatments were used to measure the expression levels of four genes. The x-axis represents the different comparisons, and the y-axis represents the RPKM values. Note: A-VS-B, blue bar represents A, red bar represents B

We also used transcriptome data from dehydration treatment [30], in which pear seedlings were dehydrated for 0 (D0), 1 (D1), 3 (D3) and 6 (D6) hours at 26 °C, followed by recovery in water at 26 °C for 24 h (DH24), to validate these seven candidate stress-related genes. The results showed that four of the seven genes were differentially expressed in two or more comparisons from five libraries. As shown in Fig. 9, the gene *Pbr4ERF24* was differentially expressed in nine comparisons, and its expression level peaked when the seedlings were dehydrated for six hours. The gene *Pbr1ERF9* was differentially expressed in six comparisons, D0-VS-D3, D0-VS-D6, D1-VS-D6, D3-VS-D6, D3-VS-DH24, and D6-VS-DH24. Its gene expression peak also appeared at six hours of dehydration treatment. The gene *Pbr2ERF12* was differentially expressed in six comparisons, D0-VS-D6, D0-VS-DH24, D1-VS-D6, D1-VS-DH24, D3-VS-D6, and D3-VS-DH24, while the gene *Pbr7ERF63* was differentially expressed only in the D0-VS-D1 and D1-VS-DH24 comparison. These results strongly supported the involvement of *ERF* genes in the response to drought stress in pear.

## Discussion

As an important plant-specific transcription factor, the AP2/ERF gene family has been widely studied in many plants, such as *Arabidopsis* [14], poplar [15], grape [16], and rice [17]. However, relatively few pear *ERF* genes were investigated in previous studies. Herein, 191 members of the AP2/ERF superfamily were identified from pear genome, including 155 members from the ERF family. Previous studies have showed that similar numbers of members have been identified in plant species, a total of 202 members of AP2/ERF superfamily including 169 members of ERF family in poplar, and 180 members of AP2/ERF superfamily including 145 members of ERF family in rice. Meanwhile, these three species have the similar genome size, 527 Mb in pear, 480 Mb in poplar and 466 Mb in rice, indicating that the number of members in AP2/ERF superfamily were closely related to genome size in the three species. In the present study, some unanchored ERF genes and genes with incomplete domain were removed. Although this decreased the potential number of members in ERF family of pear, it made the candidate ERF genes more reliable.

Most of *PbrERF* genes were duplicated and retained from WGD event, and the extensive dispersed duplication was found in pear ERF family. A recent lineage-specific WGD event (30~ 45 MYA) [21] has likely contributed to the higher proportions of WGD-type *PbrERF* gene duplications observed in pear. Meanwhile, it also indicated that WGD events played major roles in the evolution and expansion of the *PbrERF* gene family in pear. Interestingly, previous analyses showed that recent gene duplication appears to be involved in the expansion of ERF family in apple [22], further supporting similar expansion patterns of the ERF family in both pear and apple. The calculation of Ks of ERF gene pairs further supported that most of *ERF* genes duplicated from the same recent and ancient WGD events. In addition, higher Ks values (1.92–3.37) were found for six (8.00%) duplicated gene pairs, and lower Ks values (0.004–0.01) were found for four (5.33%) gene pairs (Additional file 4), which suggested that other duplication events have occurred to drive the evolution of the ERF family in pear.

Phylogenetic tree of full-length amino acid sequences of ERF in pear and *Arabidopsis* revealed that most of subgroups in ERF family included both of genes from pear and *Arabidopsis*, indicating these *ERF* genes pre-date the species divergence. Genes in the same subgroups share the similar gene structures, which decide the similar gene functions. Therefore, comparison with known function of *ERF* genes in *Arabidopsis* could help to identify candidate orthologous genes of pear and predict their gene functions. As previous study has reported that most of subgroups in *Arabidopsis* were predicted involving in plant growth and stress responses [22], a plant without fruit. It is relatively limited to use *Arabidopsis ERF* genes as the queries in phylogenetic tree to identify the functional genes in pear fruit growth and development. However, RNA-Seq data provided us a complete expression profile of *ERF* genes in different fruit developmental stages, which showed at least one gene, in all subgroups, highly expressed at all stages. Gene expression can also provide us important clues to perform the gene function prediction [59]. The function prediction analysis indicated most of the *AP2/ERF* gene family members to be involved in abiotic and/or biotic stress response. Therefore, we predicted that the genes of each group that were highly expressed throughout growth and development might play more important roles in stress response. qRT-PCR analysis showed that seven genes in group III indeed differently expressed between wild and cultivated pears. Furthermore, transcriptome data from dehydration treatment [23] validated that four of seven *PbrERF* genes were differentially expressed in different treatments, while the remaining three genes might play more important roles in other stress responses.

## Conclusion

This is the first comprehensive study on *AP2/ERF* gene family in pear aiming to help clarifying the gene function, evolution and expression pattern. The *AP2/ERF* gene family is greatly expanded in pear, and WGD event plays the important role. Function predication and expression divergence between duplicated genes revealed that the *ERF* genes are involved in multiple regulation pathways, multiple evidence supported that genes in group III might be involved in responses to abiotic stress. These results will lay a valuable foundation to understand the function and evolution of the *ERF* gene family in pear and other related species.

## Additional files

Additional file 1: The seven pairs of primer sequences for amplifying functional genes in group III for qRT-PCR analysis (XLS 36 kb)

Additional file 2: The gene name, gene ID, domain description, start position, end position, chromosome, and gene length of 191 pear *AP2/ERF* genes. (XLS 60 kb)

Additional file 3: The duplication event types of 155 pear *ERF* genes. (XLS 54 kb)

Additional file 4: The Ka, Ks, and Ka/Ks values of 75 pear *ERF* gene pairs. (XLS 42 kb)

Additional file 5: The orthologous relationships between pear and *Arabidopsis ERF* genes. (XLS 49 kb)

Additional file 6: The RPKM (reads per kilobase per million) values for *PbrAP2/ERF* gene expression. (XLS 98 kb)
